# Association between school-based physical activity promotion and mental health: a population-based cross-sectional study of schoolchildren in Finland

**DOI:** 10.1093/eurpub/ckag020

**Published:** 2026-02-10

**Authors:** Tia Viskari, Kaija Appelqvist-Schmidlechner, Timo Ståhl, Jani P Vaara, Sari Fröjd

**Affiliations:** Unit of Health Sciences, Faculty of Social Sciences, Tampere University, Tampere, Finland; Department of Public Health and Welfare, Finnish Institute for Health and Welfare, Helsinki, Finland; Department of Public Health and Welfare, Finnish Institute for Health and Welfare, Helsinki, Finland; Department of Public Health and Welfare, Finnish Institute for Health and Welfare, Helsinki, Finland; Department of Leadership and Military Pedagogy, National Defence University, Helsinki, Finland; Unit of Health Sciences, Faculty of Social Sciences, Tampere University, Tampere, Finland

## Abstract

Growing evidence emphasizes the crucial role of schools in promoting both physical activity (PA) and mental health. This study examined the associations of school-based PA promotion measures with mental health among schoolchildren in Finland. The data were extracted from two sources: the Finnish School Health Promotion study providing individual-level data and the Finnish Benchmarking System for Health Promotion Capacity Building providing school-level data. The combined dataset comprised 87 372 schoolchildren from 1662 schools. The PA promotion efforts were measured in four categories. Mental health outcomes included depressive and anxiety symptoms, self-esteem, and prosocial behaviour. Multilevel binary logistic regression was used as a statistical method. In adjusted analyses, enabling the utilization of indoor sports facilities outside of physical education (PE) classes was associated with lower likelihood of low self-esteem (odds ratio [OR]: 0.93, 95% confidence interval [CI]: 0.87–0.99). Furthermore, extended PA breaks were associated with lower likelihood of low self-esteem among boys (OR: 0.91, 95% CI: 0.83–0.99), and enabling the utilization of indoor sports facilities outside of PE classes was associated with a lower likelihood of depressive symptoms among girls (OR: 0.93, 95% CI: 0.87–0.99). The simultaneous implementation of all four PA promotion measures was associated with better prosocial skills (OR: 1.06, 95% CI: 1.01–1.11). School-based PA promotion may enhance children’s self-esteem and prosocial skills, and in girls reduce the risk of depressive symptoms, although the observed associations were particularly weak. To support the mental health of schoolchildren, PA should be promoted in schools through a variety of approaches.

## Introduction

Mental health problems are currently pressing concerns regarding the health of children and adolescents globally. The prevalence of mental health disorders is estimated to be around 8% among children aged 5–9, with the proportion increasing to 14% among adolescents aged 10–19 [1]. However, mental health encompasses not only the absence of problems but also mental well-being [1]. Mental well-being is regarded as a valuable resource, encompassing subjective well-being, effective functioning in life, and positive interactions with other people [1, 2].

One approach to promote the mental health of children and adolescents is through physical activity (PA), as the benefits for mental health are well-known [3, 4]. Schools are ideal settings for promoting PA [5] and mental health [1, 6], as they reach 91% of children globally [7]. In the Finnish education system, basic education, including primary and lower secondary school, is compulsory for all children. Basic education comprises grades 1–9 and is intended for all children aged 7–17. Basic education is free of charge, and the schools are generally maintained by the municipalities [8]. According to national norms, children have 2–3 lessons (45 minutes each) of physical education (PE) per week [9]. The roots of promoting PA beyond PE in Finnish schools lie in the Finnish Schools on the Move programme [10]. Through this initiative, municipalities received state funding to support PA promotion between 2016 and 2018. At the time of conducting the study, there were no specific requirements or guidelines for promoting PA in Finnish schools; the responsibility for PA promotion lied with individual schools and teachers. Most schools have a gymnasium and plenty of sports equipment, which are primarily used for PE classes. Finnish school days typically include several 15-minute breaks and often one longer break during the day, which are spent mainly outdoors regardless of the weather. Breaks provide additional opportunities for PA, and in most schools, children can borrow some sports equipment. However, typically there is no guided PA during breaks, and children are not required to be physically active; nevertheless, the use of mobile phones is prohibited.

Previous research has shown that school-based PA promotion supports mental health, as it has been shown to reduce anxiety [11], increase mental well-being [11, 12], and promote the social well-being of pupils [13, 14]. However, the evidence from the intervention studies is still partly contradictory [15] and incomplete [12], and the studies have mainly focused on short-term PA interventions. Therefore, more research is needed on the potential of sustained efforts to promote PA in schools in order to enhance the mental health of schoolchildren. The aim of this study is to examine the associations of school-based PA promotion measures with mental health among Finnish fourth- and fifth-grade children. In addition, the study investigates potential gender differences in these associations.

## Methods

### Data and participants

The study employed a cross-sectional design, extracting data from two sources: the Finnish School Health Promotion study (SHP) and the Finnish Benchmarking System for Health Promotion Capacity Building (BSHPCB).

The SHP study has been monitoring the health and well-being of Finnish children and adolescents since 1996. The nationwide data collection is conducted every second year via anonymous and voluntary classroom administered questionnaires for all fourth-, fifth-, eighth-, and ninth-grade pupils. In this study, we used the data of fourth- and fifth-graders (aged 10–12 years) from 2023. The study included 83% of all Finnish fourth- and fifth-graders (*n* = 102 866). The procedure of the SHP study has been approved by the ethics committee of the Finnish Institute for Health and Welfare [16]. The SHP study provided data on the mental health outcomes and background factors of study participants at the individual level.

The BSHPCB is a Finnish nationwide benchmarking tool assessing structures, capacities, and measures taken to develop health promotion practices at the local level since 2009. The BSHPCB framework measures health promotion capacity building in seven dimensions: commitment, management, monitoring and needs assessment, resources, common practices, participation, and other core functions. The BSHPCB data are collected from all Finnish municipalities every second year using an electronic form in seven sectors of administration: municipal management, basic education, upper secondary education, vocational education, PA, health and well-being services, and culture [17]. In basic education, the data collection form is sent to all Finnish basic education schools, and it is usually completed by the school principal in collaboration with pupil welfare services. In this study, we used the BSHPCB basic education data from 2023, including 93% of all basic education schools in Finland (*n* = 1930). The BSHPCB data provided information on what PA promotion measures were conducted in schools along with background information about schools.

For the present study, the data from the SHP study and the BSHPCB data were combined. Combined data comprised a total of 87 372 schoolchildren (70% of all Finnish fourth- and fifth-graders) from 1662 schools (91% of schools including grades 4 and 5 in Finland). The remaining 30% did not participate because they were absent due to various reasons (illness, travel, unauthorized absences, severe disabilities, or homeschooling) or because their schools did not participate in the BSHPCB data collection. This unique nationwide dataset enabled the investigation of the associations between school PA promotion activity and pupils’ mental health.

### Measures

#### Pupil data

The mental health and background information of study participants were measured using the following instruments.

*Depressive symptoms* were measured using the validated Finnish short version of the Mood and Feelings Questionnaire (FsMFQ-6). FsMFQ-6 is a self-reported instrument including six statements, with the response options ‘True’ (1 point), ‘Sometimes’ (0 points), and ‘Not true’ (0 points) [18]. In the dichotomized variable, *depressive symptoms* were recorded as ‘Yes’ if the respondent scored at least one point, and ‘No’ otherwise [19].

*Anxiety symptoms* were measured using five statements from the validated, self-reported Screen for Child Anxiety Related Emotional Disorders (SCARED), with the response options ‘Very true or often true’ (2 points), ‘Somewhat true or sometimes true’ (1 point), and ‘Not true or hardly ever true’ (0 points) [20]. The selected statements include one statement from each of the five SCARED subscales. The selection of the statements was based on an epidemiological study [21] so that the statements with the highest sensitivity for predicting possible anxiety disorder were chosen [22]. In the dichotomized variable, *anxiety symptoms* were recorded as ‘Yes’ if the respondent’s total score for the five SCARED statements was at least three points or the respondent had selected at least one ‘Very true or often true’ option in the questionnaire. Otherwise, anxiety symptoms were recorded as ‘No’ [19].

*Self-esteem* was measured with the positive factor of the validated, self-reported Rosenberg Self-Esteem Scale (RSES) [23]. The positive factor of the RSES includes five statements, with the response options ‘Strongly agree’ (4 points), ‘Agree’ (3 points), ‘Disagree’ (2 points), and ‘Strongly disagree’ (1 point). In the dichotomized variable, *low self-esteem* was recorded as ‘Yes’ if the respondent’s average score for the five questions was below 2.5, and ‘No’ otherwise [19].

*Prosocial behaviour* was measured using four statements from the validated, self-reported Multisource Assessment of Social Competence Scale (MASCS), with the response options ‘Very frequently’ (4 points), ‘Frequently’ (3 points), ‘Rarely’ (2 points), and ‘Never’ (1 point) [24]. In the dichotomized variable, *good prosocial skills* were recorded as ‘Yes’ if the respondent scored at least 14 points in total, and ‘No’ otherwise [19].

*Gender* was measured using a question ‘What is your official gender?’ with the response options ‘Boy’ or ‘Girl’.

*Financial situation of the family* was measured through the question ‘Which of these do you have?’ with four statements: ‘Some clothes that were bought new’, ‘Usually enough food at home’, ‘Your own phone’, and ‘Money for small personal expenses’. The response options were ‘Yes’, ‘No, my family can’t afford that’, or ‘Never, for other reasons’. The *financial situation of the family* was recorded as ‘Low’ if at least one statement was answered with ‘No, my family can’t afford that’ option or else as ‘At least moderate’.

*Family structure* was measured using the question ‘Where do you live? Select the option that best describes your situation’. There were nine response options: ‘In a shared home with my parents’, ‘I live roughly for the same length of time with both parents, who do not live together, for example, on alternative weeks’, ‘I mainly live with one of my parents and stay with the other parent from time to time, for example, at weekends’, ‘With one of my parents’, ‘With my grandparents or other relatives, without my parents’, ‘In a foster family’, ‘At a children’s home, a youth home, or a reform school’, ‘In a professional foster home’, and ‘Somewhere else’. The responses were dichotomized as ‘Nuclear family’ (option ‘In a shared home with my parents’) or ‘Other’.

#### School data

*School-based PA promotion* was measured using the question ‘Which of the following measures to increase PA during the school day have been implemented at your school?’: (1) ‘Indoor sports facilities are utilized outside of PE classes during the school day’, (2) ‘Pupils have been encouraged to engage in active commuting to school’, (3) ‘The school has extended PA breaks’, and (4) ‘Pupils have been trained as peer instructors for PA’. The response options were ‘Yes’ or ‘No’.

Utilizing indoor sports facilities outside of PE classes during school days in a Finnish school can typically mean, for example, the opportunity to spend one of the day’s breaks in the school gymnasium being active or using indoor sports facilities or equipment for functional learning in subjects other than PE. Encouraging active commuting typically means motivating children and parents to engage children in commuting to school by cycling or walking. An extended PA break typically means a 20–30 minute break during which pupils can borrow outdoor sports equipment and use the playground’s sports facilities. Pupils are encouraged to be active during breaks, but participation is usually voluntary, and there is typically no guided PA during these breaks. Trained peer instructors typically lead short PA sessions for other pupils, for example, during morning assemblies or breaks, or they may act as break-time activity leaders for their own class, conducting brief exercise sessions at the teacher’s request during lessons.

*School size* was described as the total number of pupils.

### Statistical analyses

Individual and school data were merged into a single dataset for analysis. As the mental health outcome variables were nonnormally distributed, they were dichotomized for the purpose of analyses. Dichotomization was performed using the scoring developed by the experts of the SHP study [19]. Descriptive statistics were presented with proportions for individual and school data. Gender differences in the mental health outcomes were tested using the chi-square test. Multilevel binary logistic regression nesting pupils within schools was used to test the associations between school PA promotion measures and dichotomized mental health outcomes. First, the associations between the implementation of each PA promotion measure and mental health outcomes were analysed. The reference category in these analyses was the absence of the respective PA promotion measure in the school. Analyses included only cases who had completed all questions related to the mental health outcome of interest and whose schools had provided information on the corresponding PA promotion measure. The proportions of pupils and schools with missing data are shown in Tables 1 and 2. Next, we examined whether the simultaneous implementation of all four PA promotion measures was associated with mental health outcomes. The reference category in these analyses was the implementation of less than four PA promotion measures in the school. In the latter analyses, only those schools with responses to all four statements regarding PA promotion measures were included. All analyses were conducted both unadjusted and adjusted for gender, financial situation of the family, family structure, and school size, as these variables have been suggested to be associated with children’s mental health [25–28]. Gender-specific subgroup analyses were performed as unadjusted analyses and adjusted analyses including the financial situation of the family, family structure, and school size.

**Table 1.** Descriptive characteristics of pupils from the SHP study 2023

	Boys, *n* (%) (*n* = 43 093)		Girls, *n* (%) (*n* = 43 928)		Total, *n* (%) (*n* = 87 372)	
School grade						
Fourth grade	21 260	(49.3)	21 691	(49.4)	43 067	(49.3)
Fifth grade	21 749	(50.5)	22 166	(50.5)	44 043	(50.4)
Missing	84	(0.2)	71	(0.2)	262	(0.3)
Financial situation of the family						
Low	1127	(2.6)	1307	(3.0)	2443	(2.8)
At least moderate	40 604	(94.2)	41 626	(94.8)	82 538	(94.5)
Missing	1362	(3.2)	995	(2.3)	2391	(2.7)
Family structure						
Nuclear family	31 108	(72.2)	31 225	(71.1)	62 549	(71.6)
Other	11 201	(26.0)	12 315	(28.0)	23 618	(27.0)
Missing	784	(1.8)	388	(0.9)	1205	(1.4)
Depressive symptoms						
Yes	7698	(16.4)	11 947	(24.9)	19 723	(20.6)
No	34 116	(72.5)	30 915	(61.3)	65 240	(68.3)
Missing	5236	(11.1)	5214	(10.8)	10 560	(11.1)
Anxiety symptoms						
Yes	7542	(16.0)	15 364	(32.0)	23 014	(24.1)
No	34 284	(72.9)	27 644	(57.5)	62 110	(65.0)
Missing	5224	(11.1)	5068	(10.5)	10 399	(10.9)
Low self-esteem						
Yes	3450	(7.3)	7553	(15.7)	11 059	(11.6)
No	37 628	(80.0)	34 230	(71.2)	72 076	(75.5)
Missing	5972	(12.7)	6293	(13.1)	12 388	(13.0)
Good prosocial skills						
Yes	12 046	(25.6)	17 574	(36.6)	29 700	(31.1)
No	29 808	(63.4)	25 410	(52.9)	55 425	(58.0)
Missing	5196	(11.0)	5092	(10.6)	10 398	(10.9)

n: sample size; SHP: School Health Promotion study.

**Table 2.** The implementation of physical activity promotion in schools (*n* = 1662) within the BSHPCB 2023 data

	Yes *n* (%)	No *n* (%)	Missing *n* (%)
Indoor sports facilities are utilized outside of physical education classes during the school day	1274 (76.7)	369 (22.2)	19 (1.1)
Pupils have been encouraged to engage in active commuting to school	1186 (71.4)	458 (27.6)	18 (1.1)
The school has extended physical activity breaks	1229 (73.9)	412 (24.8)	21 (1.3)
Pupils have been trained as peer instructors for physical activity	901 (54.2)	739 (44.5)	22 (1.3)

BSHPCB: Finnish Benchmarking System for Health Promotion Capacity Building; n: sample size.

The level of statistical significance was set to *P *< .05. All statistical analyses were performed using IBM SPSS Statistics version 29.0.2.0.

## Results

Table 1 presents the descriptive characteristics of the study population. The proportions of both genders and both grade levels were equal. Most of the pupils had at least a moderate financial situation in their family (94.5%) and were living in a nuclear family (71.6%). Girls experienced depressive (24.9%) and anxiety symptoms (32.0%) and had low self-esteem (15.7%) more frequently (*P *< .001 in all analyses) than boys (16.4%, 16.0%, and 7.3%, respectively). On the other hand, girls were more likely (*P *< .001) to report good prosocial skills (36.6%) in comparison to boys (25.6%) (Table 1).

The number of pupils in the schools ranged from 6 to 1479, with a median of 180. Utilizing indoor sports facilities outside of PE classes during the school day, encouraging pupils’ active commuting, and extended PA breaks were quite common in the schools, as each was reported to be implemented in over 70% of the schools. Training peer instructors for PA was less common and was implemented in slightly more than half of the schools (Table 2). All four PA promotion measures were implemented in 29.1% of the schools.

The results of the multilevel binary logistic regression are presented in Table 3. After adjustment for gender, financial situation of the family, family structure, and school size, enabling the utilization of indoor sports facilities was associated with lower likelihood of low self-esteem (odds ratio [OR]: 0.93, 95% confidence interval [CI]: 0.87–0.99). The higher likelihood of good prosocial skills was significantly associated with having peer instructors for PA in an unadjusted analysis (OR: 1.05, 95% CI: 1.01–1.10), but the association attenuated to non-significant after adjustments (OR: 1.04, 95% CI: 0.99–1.09). Further, encouraging pupils to engage in active commuting significantly increased the likelihood of good prosocial skills in the adjusted model (OR: 1.06, 95% CI: 1.01–1.11). In gender-specific analyses, there was a lower likelihood of low self-esteem among boys if the school had extended PA breaks (OR: 0.91, 95% CI: 0.83–0.99). Among girls, there was a lower likelihood of depressive symptoms (OR: 0.93, 95% CI: 0.87–0.99), anxiety symptoms (OR: 0.94, 95% CI: 0.88–0.99), and low self-esteem (OR: 0.92, 95% CI: 0.85–0.99) if the school utilized indoor sports facilities. However, only the association between depressive symptoms and utilizing indoor sports facilities (OR: 0.93, 95% CI: 0.87–0.99) remained statistically significant in the adjusted analyses. In addition, a higher likelihood of good prosocial skills in the schools implementing all four PA promotion measures was observed in the adjusted model across the entire study population (OR: 1.06, 95% CI: 1.01–1.11) as well as among girls (OR: 1.07, 95% CI: 1.01–1.13), compared to schools implementing fewer than four measures.

**Table 3.** Physical activity promotion measures of the school and mental health outcomes among study participants

	Non-adjusted			Adjusted		
	Boys OR (95% CI)	Girls OR (95% CI)	Total OR (95% CI)	Boys^a^ OR (95% CI)	Girls^a^ OR (95% CI)	Total^b^ OR (95% CI)
Depressive symptoms						
Indoor sports facilities^c^	0.99 (0.92–1.07)	0.93 (0.87–0.99)	0.96 (0.91–1.01)	0.99 (0.92–1.07)	0.93 (0.87–0.99)	0.96 (0.90–1.01)
Active commuting to school^c^	1.02 (0.95–1.10)	1.00 (0.94–1.06)	1.00 (0.95–1.05)	1.00 (0.93–1.08)	1.00 (0.94–1.07)	1.00 (0.95–1.06)
Extended PA breaks^c^	1.02 (0.94–1.10)	0.99 (0.92–1.05)	1.01 (0.95–1.06)	1.02 (0.95–1.10)	0.98 (0.91–1.05)	1.00 (0.95–1.06)
Peer instructors for PA^c^	1.01 (0.95–1.07)	0.96 (0.92–1.01)	1.00 (0.96–1.05)	1.00 (0.94–1.07)	1.00 (0.94–1.06)	1.00 (0.95–1.05)
All four PA promotion measures^d^	1.05 (0.99–1.12)	0.99 (0.93–1.04)	1.02 (0.97–1.06)	1.04 (0.98–1.11)	0.98 (0.93–1.04)	1.01 (0.96–1.06)
Anxiety symptoms						
Indoor sports facilities^c^	1.02 (0.95–1.10)	0.94 (0.88–0.99)	0.97 (0.92–1.02)	1.03 (0.96–1.11)	0.95 (0.89–1.01)	0.98 (0.93–1.03)
Active commuting to school^c^	1.02 (0.95–1.10)	0.99 (0.94–1.05)	1.00 (0.96–1.05)	1.03 (0.96–1.10)	1.00 (0.94–1.05)	1.01 (0.96–1.06)
Extended PA breaks^c^	0.97 (0.90–1.04)	0.97 (0.91–1.03)	0.98 (0.93–1.03)	0.97 (0.90–1.05)	0.97 (0.91–1.03)	0.97 (0.92–1.02)
Peer instructors for PA^c^	0.96 (0.90–1.02)	0.96 (0.92–1.01)	0.97 (0.93–1.01)	0.96 (0.90–1.02)	0.97 (0.92–1.02)	0.96 (0.92–1.01)
All four PA promotion measures^d^	0.99 (0.93–1.05)	0.98 (0.93–1.03)	0.99 (0.95–1.03)	0.99 (0.92–1.05)	0.98 (0.93–1.03)	0.98 (0.94–1.03)
Low self-esteem						
Indoor sports facilities^c^	0.92 (0.85–1.00)	0.92 (0.85–0.99)	0.92 (0.86–0.98)	0.94 (0.86–1.02)	0.93 (0.86–1.00)	0.93 (0.87–0.99)
Active commuting to school^c^	0.95 (0.87–1.03)	0.97 (0.90–1.04)	0.95 (0.89–1.01)	0.94 (0.87–1.02)	0.97 (0.90–1.05)	0.95 (0.90–1.02)
Extended PA breaks^c^	0.91 (0.84–0.99)	0.98 (0.91–1.06)	0.96 (0.90–1.02)	0.91 (0.83–0.99)	0.97 (0.90–1.05)	0.94 (0.88–1.01)
Peer instructors for PA^c^	0.99 (0.92–1.06)	1.01 (0.94–1.07)	1.00 (0.94–1.05)	0.99 (0.92–1.07)	1.02 (0.95–1.09)	1.01 (0.95–1.07)
All four PA promotion measures^d^	0.95 (0.89–1.02)	0.97 (0.91–1.04)	0.96 (0.91–1.01)	0.94 (0.88–1.02)	0.97 (0.91–1.04)	0.96 (0.90–1.01)
Good prosocial skills						
Indoor sports facilities^c^	0.96 (0.89–1.02)	1.00 (0.94–1.07)	0.97 (0.92–1.03)	0.95 (0.89–1.02)	0.99 (0.93–1.06)	0.97 (0.91–1.02)
Active commuting to school^c^	1.04 (0.98–1.11)	1.06 (1.00–1.13)	1.05 (1.00–1.11)	1.05 (0.98–1.12)	1.06 (0.99–1.12)	1.06 (1.01–1.11)
Extended PA breaks^c^	1.05 (0.98–1.12)	1.01 (0.95–1.08)	1.03 (0.97–1.09)	1.04 (0.97–1.11)	1.01 (0.95–1.08)	1.02 (0.96–1.08)
Peer instructors for PA^c^	1.04 (0.98–1.10)	0.99 (0.89–1.11)	1.05 (1.01–1.10)	1.03 (0.97–1.09)	1.05 (0.99–1.11)	1.04 (0.99–1.09)
All four PA promotion measures^d^	1.05 (0.99–1.11)	1.08 (1.02–1.14)	1.06 (1.01–1.11)	1.04 (0.98–1.11)	1.07 (1.01–1.13)	1.06 (1.01–1.11)

a Adjusted to include financial situation of the family, family structure, and school size.

b Adjusted to include gender, financial situation of the family, family structure, and school size.

c Reference category: the absence of a PA promotion measure.

d Reference category: fewer than four PA promotion measures.

CI: confidence interval; OR: odds ratio; PA: physical activity.

## Discussion

This study examined the associations between school-based PA promotion measures and mental health among Finnish schoolchildren. We found that enabling the utilization of indoor sports facilities during the school day was associated with stronger self-esteem. Furthermore, the simultaneous implementation of all four examined PA promotion measures was associated with better prosocial skills. The findings also revealed gender-specific differences between different PA promotion means and selected mental health outcomes.

Enabling the utilization of indoor sports facilities during the school day beyond PE classes was associated with stronger self-esteem. This aligns with the findings of a previous meta-analysis, which found that PA was associated with increased self-concept and self-worth in children and adolescents, with stronger associations observed in school/gymnasium-based interventions compared to other settings [29]. The benefits of utilizing indoor sports facilities may be attributed to the diverse PA opportunities they offer throughout the year. Cold and wet weather conditions have been found to be associated with reduced PA levels in children and adolescents [30]. In Finland, most of the school year consists of autumn and winter, which is commonly dark, cold, wet, and often slippery. Children wear thick and heavy winter clothing and footwear, which particularly restricts vigorous play and games. Slipperiness limits the practice of many sports outdoors during winter as well. Indoor facilities thus provide an opportunity for diverse and vigorous PA regardless of weather conditions. Improvement in perceived sport competence has been suggested as a potential mechanism underlying the relationship between PA and self-esteem in children and adolescents [31].

Subgroup analyses revealed that extended PA breaks were associated with stronger self-esteem among boys. Previous studies have found that boys are more active during breaks compared to girls [32]. This may be because schoolyards tend to be more appealing for PA for boys than girls. Ball games are the most popular form of PA among Finnish elementary school boys [33]. In Finnish schools, breaks are spent outdoors, and schoolyards typically offer opportunities for various ball games. Among girls, the reporting of depressive symptoms was less frequent when the utilization of indoor sports facilities was enabled during the school day. The result is consistent with previous research findings that found PA reduces depressive symptoms in children and adolescents [34]. In this study, the association was found solely among girls, possibly because depressive symptoms were less reported among boys. Girls may benefit particularly from the use of indoor sports facilities, as they offer better opportunities for physical activities that interest girls, such as dance, gymnastics, and aerobics [33].

The simultaneous implementation of all examined PA promotion measures in school was associated with better prosocial skills. The result is consistent with previous research, as PA has been found to improve prosocial behaviour [35], and school-based PA promotion has shown to enhance social well-being [13, 14] among children and adolescents. A potential explanation for the association between PA and prosocial skills may lie in the increased levels of empathy [36], which has been identified as a driver of prosocial behaviour [37]. In addition to the possible increase in PA, the association between diverse PA promotion and prosocial skills may also be partly explained by the sense of community in the school. Promoting PA diversely requires a communal culture within the school, which has been found to be positively associated with children’s social well-being [38].

The associations between PA promotion measures and mental health were relatively weak but statistically significant. Previous research has found that school-based preventive programmes in general have only small beneficial effects on mental health [39]. However, considering the enduring and easily integrated nature of the studied PA promotion measures within the daily life of the school, the results can be considered significant. The PA promotion measures studied can be implemented with relatively modest additional resources from schools and minimal effort from teachers, without disrupting schoolwork or adherence to the curriculum, which have been identified as the main barriers to promoting PA in schools [40]. Due to the nature of these measures, they can be extended to a large population of children and adolescents, and their permanence may potentially achieve long-term benefits.

A major strength of this study lies in its unique, nationwide dataset, which comprehensively includes information on PA promotion in Finnish schools as well as the mental health of the children attending these schools. The representativeness of the sample size was very high, as 70% of all fourth- and fifth-graders and 91% of schools including grades 4 and 5 took part. In addition, the study considered mental health broadly, encompassing both positive and negative aspects of mental health. The survey was administered in the presence of a teacher with a strong focus on maintaining each pupil’s anonymity. The analyses were adjusted for factors known to confound the associations based on prior research evidence.

The study also has limitations that should be considered. First, the outcome variables are based on validated measures, but the shortened versions of the measures for anxiety symptoms, self-esteem, and prosocial behaviour used in this study have not been validated and therefore cannot be considered fully comparable to the original instruments. However, the questions were carefully selected by an expert group, and these shortened measures have become an established part of the nationwide SHP study. Second, we do not know whether the PA promotion measures reported by schools have increased children’s PA. When interpreting the results, it is important to note that the study measured whether the school enabled certain activities for pupils to be more active during the school day. However, we do not know the amount of PA among children based on these data. Finally, the cross-sectional design does not allow for conclusions about causal relationships.

Future research is needed to investigate similar associations in other age groups. Longitudinal studies and randomized controlled trials are required to draw conclusions about causal relationships. Future research should also investigate the mechanisms behind the associations.

Conflict of interest: None declared.

## Data Availability

The data that support the findings of this study are available from the Finnish Institute for Health and Welfare, but restrictions apply to the availability of these data, which were used under license for the current study, and are not publicly available. The data are available from the authors upon reasonable request and with the permission of the Finnish Institute for Health and Welfare. Key Points The existing literature increasingly highlights the vital role of schools in promoting both PA and mental health among children and adolescents. In this population-based cross-sectional study, we found that school-based PA promotion was associated with stronger self-esteem and better prosocial skills among Finnish schoolchildren, and among girls additionally with reduced risk of depressive symptoms. However, although the associations between school-based PA promotion and mental health were statistically significant, they were particularly weak. The study showed that boys and girls benefit from different PA promotion measures: for girls, the use of indoor sports facilities seems to be particularly beneficial, while boys benefit most from extended PA breaks. To support the mental health of schoolchildren, PA should be promoted in schools through a variety of approaches.
